# Lipid-Lowering Therapy in PURE Poland Cohort Study

**DOI:** 10.3390/jcm13010060

**Published:** 2023-12-22

**Authors:** Paweł Lubieniecki, Maria Wołyniec, Katarzyna Połtyn-Zaradna, Katarzyna Zatońska, Andrzej Szuba

**Affiliations:** 1 Clinical Department of Diabetology and Internal Disease, Wroclaw Medical University, Borowska Street 213, 50-556 Wroclaw, Poland; 2 Department of Social Medicine, Wroclaw Medical University, 50-345 Wrocław, Poland; maria.wolyniec@umw.edu.pl (M.W.); katarzyna.poltyn-zaradna@umw.edu.pl (K.P.-Z.); katarzyna.zatonska@umw.edu.pl (K.Z.); 3 Clinical Department of Angiology and Internal Disease, Wroclaw Medical University, Borowska Street 213, 50-556 Wroclaw, Poland; andrzej.szuba@umw.edu.pl

**Keywords:** systematic coronary risk evaluation 2, prospective urban and rural epidemiological study, cardiovascular risk, lipid-lowering therapy

## Abstract

The aim of this study is to present data on the use of lipid-lowering therapy (LLT) in relation to calculated cardiovascular risk (CVR) and an additionally defined target LDL-C concentration. The cohort consisted of 1287 participants in the Polish edition of the Prospective Urban and Rural Epidemiological Study (PURE). CVR was calculated for each participant using the SCORE2 or SCORE2-OP scale, and for patients with diabetes mellitus (DM), chronic kidney disease (CKD) or atherosclerotic cardiovascular disease (ASCVD) according to the respective criteria. In the cohort analysed, 107 of 212 people (50.5%) in the low cardiovascular risk (CVR) group, 284 of 414 people (68.6%) in the moderate CVR group, 562 of 612 people (91.8%) in the high CVR group and 48 of 49 people (98%) in the very high CVR group did not meet the target LDL-c criterion. Of those in the low CVR group, 86% of participants were not receiving lipid-lowering therapy (LLT); in the moderate CVR group, the proportion was 77.8%; in the high CVR group, 68.1% and in the very high CVR group, 75%. In each cardiovascular risk group, participants who did not meet the target LDL-c concentration criterion and did not take LLT made up the larger group.

## 1. Introduction

Cardiovascular disease (CVD) is the most common cause of death both worldwide and in Poland. In 2021, 180,760 people in Poland died from this cause, accounting for 34.8% of all such events [1]. The basis for estimating the probability of morbidity or death from this cause is the calculation of cardiovascular risk (CVR). This is a rough, estimated predictive value that determines the possibility of CVD or death from CVD over a 10-year period. Calculating an individual’s CVR is the basis for appropriate primary prevention and further identification of optimal therapy.

Currently, the European Society of Cardiology recommends using the Systematic Coronary Risk Evaluation 2 (SCORE2) scale and the Systematic Coronary Risk Evaluation 2-Older Persons (SCORE2-OP) scale to assess cardiovascular risk. This is an update of the existing SCORE scale and is based on current data on the prevalence of cardiovascular disease and its possible consequences.

It takes into account age, sex, systolic blood pressure (SBP), non-HDL cholesterol (nHDL-C) and smoking status [2,3]. It is applied to a population excluding those with atherosclerotic cardiovascular disease (ASCVD), diabetes mellitus (DM) or chronic kidney disease (CKD) with a glomerular filtration rate (eGFR) below 60 min/1.73 m^2^. Such individuals are immediately assigned to high- or very-high-risk groups for future cardiovascular incidents.

The risk calculation provides a baseline for adopting an appropriate individual strategy for each patient. The basis should be the improvement of modifiable factors, such as weight reduction, increased physical activity, smoking cessation, conservative or pharmacological treatment of hypertension or hyperlipidemia [4,5]. The basis of protective measures should be adequate patient education, including public awareness of the basics of healthy eating or proper physical activity.

In cases of dyslipidemia, lipid-lowering therapy (LLT) is used for secondary prevention. There are a number of studies showing a significant effect of statins and other hypolipemic drugs in reducing cardiovascular events [6,7,8].

We analysed a group of participants in the Polish cohort of the Prospective Urban and Rural Epidemiologic Study (PURE), which looked at CVD risk factors in residents of Wroclaw and the surrounding countryside. An individual CVR was determined for each participant, and it was determined whether they should be taking LLT according to current guidelines and whether they were actually doing so. Adequate risk identification at each stage of patient care can improve quality of life and prevent premature cardiac deaths as well as the higher expenses associated with treating CVD complications. The results are presented below.

## 2. Materials and Methods

Baseline data were collected between 2019 and 2022 and were part of the Global Prospective Urban and Rural Epidemiological Study (PURE), which has been ongoing since 2007, with data collected at 3-year intervals. All participants were screened according to the PURE study protocol [9,10], which included a questionnaire survey (individual health, household, family, food frequency questionnaire and International Physical Activity Questionnaire (IPAQ)), anthropometric measurements, blood pressure measurement, blood draw, ECG and spirometry. The baseline cohort consisted of 2035 adult participants (1281 women and 754 men) aged 30–85 years (mean: 55 years, SD ± 10). The study group included participants from urban areas (the city of Wroclaw) and rural areas (the villages surrounding Wroclaw) in Lower Silesia.

Diabetes was determined by self-reported diabetes and/or self-reported use of antidiabetic medications and/or measurement of fasting blood glucose ≥ 126 mg/dL [11]. Hypertension was determined by self-reported hypertension and/or self-reported antihypertensive medications and/or the mean of two blood pressure measurements ≥ 140/90 mmHg [12]. The presence of cardiovascular disease (CVD) and respiratory disease was reported by participants. The CVD category included participants who reported heart failure, coronary artery disease and other heart diseases. Attitudes toward tobacco smoking were self-reported by the participants. In the case of tobacco smoking, participants could have chosen one of three possible answers: “formerly used tobacco products”, “currently use tobacco products”, or “never used tobacco products”.

Lipid disorders and use of LLT were self-reported by participants. The individual health questionnaire had four questions directly related to this:

(1) Do you have high cholesterol?
(2) Are you taking medications regularly to lower your cholesterol?
(3) In the last 12 months, were you taking medications for lowering cholesterol but then stopped?
(4) In the past month, how often did you take your cholesterol medications as the doctor prescribed?

As for question three, the participant could choose from the following reasons:

(1) Doctor advised me to stop because cholesterol was under control; Felt unwell from cholesterol medication(s) so was told to stop; Felt well, no need to take my medications.
(2) Self-decision to stop because cholesterol was under control; Felt unwell from cholesterol medications so decided to stop; Felt well, no need to take my medications; Cannot afford cholesterol medications; The pharmacy is too far away from me; I have to take too many medications; My cholesterol medication is often not available in my pharmacy.

The study was reviewed and approved by the Bioethics Committee of the Medical University of Wrocław on 6. October 2006 and was therefore conducted in accordance with the ethical standards set out in the relevant version of the 1964 Declaration of Helsinki (positive opinion of the Bioethics Committee of the Medical University of Wrocław No. KB-443/2006).

The SCORE2 and SCORE2-OP scales were calibrated against four country categories, which were determined from national CVD mortality rates published by WHO [13]. Poland was classified as a high-risk country. An individual CVR for each patient was then calculated based on age, sex, smoking status, nHDL-c concentration and SBP; the participant was then assigned to one of four age- and value-appropriate categories—low-, moderate-, high- or very-high-risk—according to the SCORE2 and SCORE2-OP tables applicable to high-risk countries [2,3].

This CVR assessment scheme is not applicable to patients with ASCVD, DM and CKD. These individuals fall into one of three risk categories—moderate, high or very high [14]—according to the following findings:

I Patients with CKD without DM or ASCVD:

- High for moderate CKD (eGFR 30–44 mL/min/1.73 m^2^ and ACR (albumin-to-creatinine ratio) < 30 or eGFR 45–59 mL/min/1.73 m^2^ and ACR 30–300 or eGFR > 60 mL/min/1.73 m^2^ and ACR > 300).
- Very high for severe CKD (eGFR < 30 mL/min/1.73 m^2^ or eGFR 30–44 mL/min/1.73 m^2^ and ACR > 30).

II Patients with DM 2 and patients with DM 1 over 40 years of age [15]:

- Moderate for well-controlled, short-lived diabetes (<10 years) without data indicating TOD (target organ damage) and without additional ASCVD risk factors.
- High for patients with DM without ASCVD and/or severe TOD [16] and do not meet criteria for moderate risk.
- Very high for DM patients with ASCVD and/or severe TOD or eGFR < 45 mL/min/1.73 m^2^ regardless of albuminuria or eGFR 45–59 mL/min/1.73 m^2^ and microalbuminuria (ACR 30–300 mg/g) or proteinuria (ACR > 300 mg/g) or presence of microvascular disease in at least 3 locations.

III Patients with diagnosed ASCD:

- Very high for documented ASCVD, either clinically or unequivocally on imaging studies. Clinically documented ASCVD includes previous AMI (acute myocardial infarction), ACS (acute coronary syndrome), coronary revascularization and other arterial revascularization procedures, stroke, TIA (transient ischemic attack), aortic aneurysm and PAD (peripheral artery disease). ASCVD found unequivocally on imaging studies includes the presence of atherosclerotic plaque found on coronary angiography or ultrasound of the carotid arteries, or on CTA (computed tomography angiography).

Statistica 13.3 (TIBCO. Software Inc., Palo Alto, CA, USA) was used for statistical analysis. The work presents the results classified as the so-called industry statistics, using both descriptive statistics (age, sex) and mathematical statistics.

## 3. Results

### 3.1. Overall Results

The analysis included 1287 participants in the PURE study. Participants with ASCVD, CKD and DM who knew the duration of the last disease—a factor necessary to qualify the patient for the appropriate CVR group—were analysed. For participants without these diseases, all participants with the full set of data necessary to calculate SCORE2 or SCORE2-OP (age, sex, smoking status, SBP and nHDL-c) were analysed. The remaining participants were excluded from this analysis. The cohort consisted of 441 men (34.7%) and 846 women (65.3%). Based on place of residence, the number of urban residents amounted to 877 (68.1%), while the number of rural residents amounted to 410 (31.9%). 

A total of 347 people (27%) reported using LLT. Of those, 30 participants had discontinued such treatment in the past 12 months, including 8 who discontinued treatment because of a physician’s decision: 7 participants due to good lipid control and 1 participant due to poor health resulting from the use of the aforementioned medications. A total of 22 participants discontinued treatment on their own: 6 due to lack of effects, 7 due to poor health resulting from the use of LLT, 7 who did not feel the need to use LLT while feeling well, and 2 who discontinued treatment due to too much medication in daily use.

When calculating CVR using the SCORE-2 and SCORE-2-OP scales, 1064 people were included: 712 women and 352 men or, taking into account place of residence, 742 urban residents and 322 rural residents. The group with DM, CKD or ASCVD included 223 people: 134 women and 89 men or, considering place of residence, 135 urban residents and 88 rural residents. Overall results are shown in Table 1.

### 3.2. Outcomes in Specific Cardiovascular Risk Groups

#### 3.2.1. Patients Classified in the Low CVR Group

Patients classified in the low CVR group comprised 212 participants (16.4%). Of these, 105 achieved the target LDL cholesterol concentration (LDL-c) (49.5%) and 107 participants did not (50.5%). A pooled analysis of the results without disaggregating patients by comorbidities is shown in Table 2. Among these 107 participants, 92 participants (86%) were not taking LLT. This group included 69 urban residents (75%) or 33 men (35.9%).

Patients with ASCVD, DM and CKD did not qualify for the low CVR group.

The group of 107 individuals without ASCVD, DM and CKD represented those who did not meet the LDL-c target criterion in the low CVR group. Of these, 15 people (14% of the subgroup) were taking LLT. A total of 92 people (86% of the subgroup) refused treatment. In the group of people meeting the target LDL-c concentration criterion, 23 people (21.9% of the subgroup) were receiving appropriate drug treatment. In contrast, 82 people (the remaining 78.1% of the subgroup) were not receiving any treatment.

#### 3.2.2. Patients with Moderate CVR

Among those with moderate CVR, of whom there were 414 (32.2%), 130 achieved the target LDL-c concentration (31.4%), while 284 did not reach the target concentration (68.6%). Of those 284 people, 221 people (77.8%) were not taking LLT. This group included 156 urban residents (70.6%) or, by sex, 85 men (38.4%).

The 111 participants with ASCVD, DM and CKD were assigned to the moderate CVR group. The target concentration was reached by 55 participants (49.5%). 

The number of participants with ASCVD, DM and CKD with moderate CVR who did not reach the target LDL-c concentration amounted to 56 (50.5%). Of those, 16 patients (28.6% of the subgroup) were using pharmacological treatment for lipid disorders. In contrast, 40 participants in the study (71.4% of the subgroup not meeting LDL target criteria) refused such treatment.

A total of 303 participants without ASCVD, DM and CKD were allocated to the moderate CVR group. The target concentration was reached by 75 participants (24.8%). 

The group without ASCVD, DM and CKD with moderate CVR who did not reach the target LDL concentration included 228 participants (75.2%). Of those, 47 participants (20.6% of the subgroup) were using pharmacological treatment for lipid disorders. In contrast, 181 participants (79.4% of the subgroup of those not meeting the target LDL-c concentration criteria) refused such treatment.

#### 3.2.3. Patients Classified in the High CVR Group

There were 612 patients (47.6%) in the high CVR group. Only 50 patients reached the target LDL-c concentration (8.2%), while up to 562 patients (91.8%) had impaired lipid metabolism. Of the 562 people who did not reach the target LDL-c concentration for a given CVR, 383 were not taking LLT (68.1%). This group included 274 urban residents (71.5%) or, by sex, 125 men (32.6%).

A total of 63 individuals with ASCVD, DM, CKD were assigned to the high CVR group. The target concentration was reached by 12 participants (19%). 

The group of those with ASCVD, DM, CKD with a high CVR who did not reach the target LDL-c concentration included 51 people (81%). Of those, 15 people (29.4% of the subgroup) were using pharmacological treatment for lipid disorders. In contrast, 36 study participants (70.6% of the subgroup of people not meeting the target LDL-c concentration criteria) refused such treatment.

A total of 549 participants were allocated to the group without ASCVD, DM and CKD with high CVR. Of those, 38 (6.9%) met the target LDL-c concentration criteria.

The group of 511 participants (93.1%) without ASCVD, DM and CKD represented those who did not meet the target LDL-c concentration criteria in the group with high CVR. Of those, 164 participants (32.1% of the subgroup) were taking LLT. In contrast, 347 people (67.9% of the subgroup) refused treatment.

#### 3.2.4. Patients Classified as Having a Very High CVR

In the case of very high CVR, out of 49 people (3.8%), as many as 48 people (98%) did not reach the target LDL-c concentration. Among those individuals, 36 people (75%) were not taking LLT—22 urban residents (61.1%) or, by gender, 19 men (50%). 

In the group of patients without comorbidities, such as ASCVD, DM and CKD, there were no patients meeting the criteria for very high CVR.

**Figure 1:**
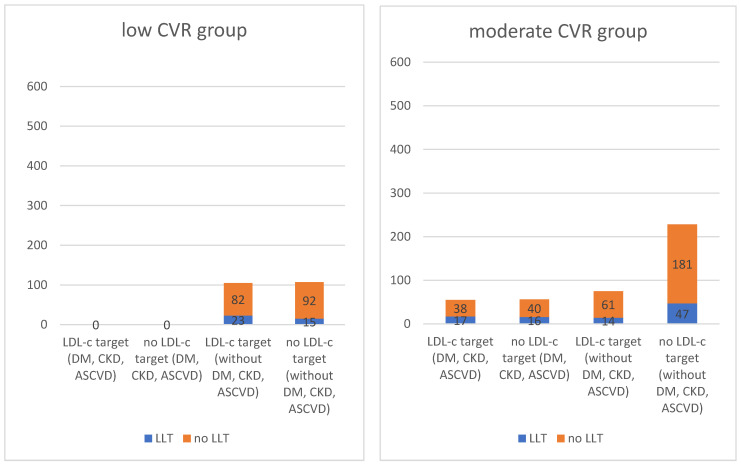

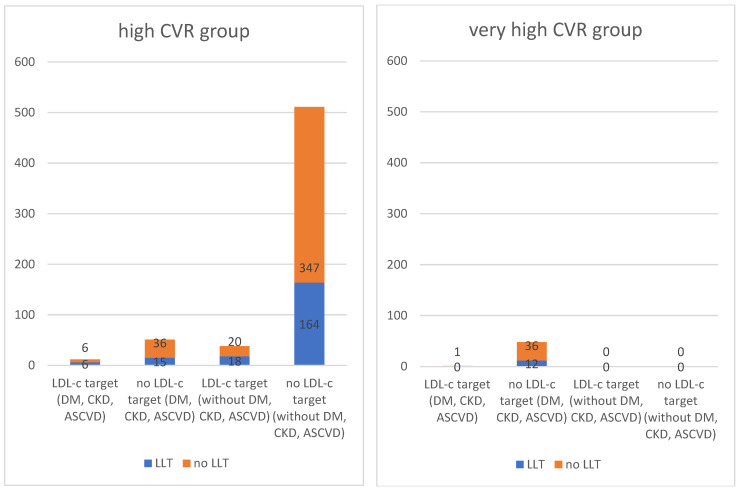
Outcomes by patients counted according to the SCORE2 scale and patients with DM, CKD and ASCVD.

Outcomes by patients counted according to the SCORE2 scale and patients with DM, CKD and ASCVD are shown in Figure 1.

## 4. Discussion

CVR assessment remains one of the primary tools for primary prevention. In August/September 2021, the European Society of Cardiology approved the SCORE2 and SCORE2-OP scales for use. They allow for estimation of an individual’s risk of cardiovascular events over the next 10 years in apparently healthy individuals. Unlike their predecessor, the SCORE scale, they calculate the risk not only of fatal episodes due to cardiovascular disease, but also of non-fatal episodes such as strokes and heart attacks. Also updated from its predecessor is the consideration of nHDL-c concentration (the difference between total cholesterol and HDL-cholesterol (HDL-c) concentration) instead of total cholesterol concentration. Due to the proatherogenic effect of apoB-containing lipoproteins, thereby contributing to the formation and progression of atherosclerotic plaques, it is important to correctly estimate their amount. The concentration of LDL cholesterol can be influenced by the concentration of triglycerides; so, according to later studies and recent guidelines, the determination of n-HDL cholesterol is preferred for the determination of CVR [17,18].

Another novelty is the approach to people over 70 years of age. Included in this population are analyses that take into account competing risks; that is, deaths from causes other than CVD. The SCORE2-OP algorithm estimates the five-year and 10-year incidence of non-fatal and fatal cardiovascular events adjusted for competing risks in apparently healthy individuals over 70 years of age [3].

Calculating an individual’s CVR is the first and most important step in primary prevention. Once the patient is classified into the appropriate group, we can adopt an individualized strategy. Starting with education, it is important to remember that a patient can be moved to a lower risk category by influencing modifiable risk factors through lifestyle changes. First and foremost, patients should be encouraged to quit smoking, as this element is one of the strongest modulators of cardiovascular disease [19]. Other aspects that can be improved include increased physical activity and proper nutrition, which reduces BMI (body mass index), which has a positive effect on CVR [20] and also has a positive effect on lipidogram profile. From a clinical point of view, one of the most important issues is the control and possible treatment of hypertension. This is a modifiable risk factor, an increase of which above established norms has a linear effect on increasing CVR [21].

Patients in the high and very high CVR groups should receive special attention and care. In our study, they accounted for more than half of all participants: 612 people (47.6%) in the high group and 49 people (3.8%) in the very high-risk group, respectively. For such individuals, pharmacological treatment of the modifiable CVD risk factors described above should be strongly considered for high and recommended for very high CVR [14]. In the absence of risk factor reduction by non-pharmacological methods, pharmacological methods, including LLT, should be considered.

In our study, 91.8% of those in the high CVR group and 98% of those in the very high CVR group did not meet the target LDL-c concentration criterion, a strong risk factor for cardiovascular disease [22,23]. Of those who did not meet the target LDL-c concentration, 68.1% of those in the high CVR group were not taking LLT. The percentage of such individuals in the high-risk group was 75%.

Pajak et al., in the WOBASZ II study, described that 60% of respondents with hypercholesterolemia were unaware of the condition, and only 6% of respondents were treated and achieved the recommended therapeutic goal. The WOBASZ II survey conducted in 2013–2014 was the second edition of the cross-sectional WOBASZ survey conducted on random samples of the Polish population in 2003–2005. One of the findings was that, relative to the first edition, the level of hypercholesterolemia remained stable and high [24].

One of the goals of the PURE study is to compare the risk factors for lifestyle diseases of urban and rural populations. Our results show that, in each risk group of people not meeting the target LDL-c concentration, urban residents were in the majority. This was 73.8% in the low CVR group, 67.6% in the moderate CVR group, 69.4% in the high CVR group and 62.5% in the very high group, respectively. One of the strongest inducers of lipid disorders is obesity. Anza-Ramirez et al. described, in their article, that residents of more densely populated cities are more likely to have increased BMI and obesity compared to those living in less urban areas [25]. Jungah et al. similarly showed a strong correlation between greater urbanization and obesity among the population in Seoul [26]. Carrillo-Larco et al. described a higher prevalence of obesity in people from urban areas and rural migrants compared to people from rural backgrounds [27].

Failure to meet the target LDL-c concentration criterion can have various causes. For those who do not require pharmacological intervention, the cause may be an abnormal lifestyle associated with an unhealthy diet. Such a group consists of people who are younger and without multimorbidity. In other cases, there is often a delay in the doctor’s inclusion of appropriate treatment. Currently, LLT is included at too late a stage. This affects the patient as an individual, as well as the overall economics of the health care system. Ciaran et al. described the significant cost-effectiveness of prophylactic statin treatment for the health care system [28].

Among those who require pharmacological intervention, the reasons for the alarmingly low percentage of patients who do not achieve target LDL levels and do not adhere to LLT may include economic considerations, lack of access to healthcare or medications, overly complicated treatment regimens, the patient’s general condition, and intentional nonadherence related to lack of faith in the effectiveness of prescribed medications or fear of their side effects. Choudry et al. described that, in a cohort of U.S. retired post-MI patients with significantly lower incomes, only 38.6% were receiving statins [29]. Nieuwerk et al. studied the effect of active counselling on adherence to statin use. Patients were randomly assigned to a standard care group and a group in which the intervention consisted of individualized, nurse-led counselling. At the end of the study, patient-reported adherence to LLT was significantly higher in the intervention group (100% vs. 95%; *p* < 0.05) [30].

Patient noncompliance is also influenced by the chronic nature of the disease and the need for long-term, largely indefinite treatment [31]. It is also necessary to take into account the diverse substrate of hyperlipidemia, the heterogeneity of this condition (including atherogenic hyperlipidemia), and thus the possible different treatment regimens [32]. Khan et al., in their article, highlighted the role of ezetimibe and PCSK9 inhibitors in reducing non-fatal myocardial infarctions and strokes [33]. Currently, these drug groups are rarely included by physicians and represent a negligible proportion of LLT.

## 5. Conclusions

In our study, we showed that 91.8% of patients in the high CVR group and 98% in the very high CVR group did not meet the target LDL-c criterion. Moreover, 68.1% of those in the first described group and 75% in the second group were not taking LLT. It is important to remember that even a small reduction in LDL-c levels in patients with high or very high CVR can improve prognosis and reduce the absolute number of vascular events in the future [34]. Greater awareness among practising physicians may lead to a greater focus on the problem and, as a result, a reduction in CVR in more people. Achieving target LDL levels while reducing CVR can be achieved by educating the patient on lifestyle changes, including diet, or, if ineffective, by incorporating LLT into treatment and then enforcing medical recommendations.

## Figures and Tables

**Table 1:** Overall results.

Polish Cohort of the PURE Study	2035
participants analysed	1287
patients with DM, ASCVD, CKD	223
other patients	1064
urban residents/rural residents	877/410
men/women	441/846
patients with low CVR	212
patients with moderate CVR	414
patients with high CVR	612
patients with very high CVR	49

**Table 2:** Summary analysis of results without dividing patients by comorbidities.

	Low CVR	Moderate CVR	High CVR	Very High CVR
number of people	212	414	612	49
urban residents	155 (73.1%)	275 (66.4%)	416 (68%)	31 (67.2%)
men	63 (29.7%)	147 (35.5%)	205 (33.55%)	26 (53.1%)
have achieved the target LDL-c	105 (49.5%)	130 (31.4%)	50 (8.2%)	1 (2%)
were taking LLT	23 (21.9%)	31 (23.8%)	24 (48%)	0 (0%)
were not taking LLT	82 (78.1%)	99 (76.2%)	26 (52%)	1 (100%)
have not achieved the target LDL-c concentration	107 (50.5%)	284 (68.6%)	562 (91.8%)	48 (98%)
were taking LLT	15 (14%)	63 (22.2%)	179 (31.9%)	12 (25%)
were not taking LLT	92 (86%)	221 (77.8%)	383 (68.1%)	36 (75%)

## Data Availability

The data are available upon request.

## References

[1] Raport: Sytuacja Zdrowotna Ludności Polski i Jej Uwarunkowania. https://www.pzh.gov.pl/raport-sytuacja-zdrowotna-ludnosci-polski-i-jej-uwarunkowania.

[2] SCORE2 Working Group and ESC Cardiovascular risk Collaboration (2021). SCORE2 risk prediction algorithms: New models to estimate 10-year risk of cardiovascular disease in Europe. Eur. Heart J..

[3] SCORE2-OP Working Group and ESC Cardiovascular Risk Collaboration (2021). SCORE2-OP risk prediction algorithms: Estimating incident cardiovascular event risk in older persons in four geographical risk regions. Eur. Heart J..

[4] Dudina A., Cooney M.T., Bacquer D.D., Backer G.D., Ducimetière P., Jousilahti P., Keil U., Menotti A., Njølstad I., Oganov R. (2011). Relationships between body mass index, cardiovascular mortality, and risk factors: A report from the SCORE investigators. Eur. J. Cardiovasc. Prev. Rehabil..

[5] Cornelius M.E., Wang T.W., Jamal A., Loretan C.G., Neff L.J. (2020). Tobacco Product Use Among Adults—United States, 2019. MMWR Morb. Mortal Wkly. Rep..

[6] Ward S., Lloyd Jones M., Pandor A., Holmes M., Ara R., Ryan A., Yeo W., Payne N. (2007). A systematic review and economic evaluation of statins for the prevention of coronary events. Health Technol. Assess..

[7] Aznaouridis K., Masoura C., Vlachopoulos C., Tousoulis D. (2019). Statins in Stroke. Curr. Med. Chem..

[8] Almeida S.O., Budoff M. (2019). Effect of statins on atherosclerotic plaque. Trends Cardiovasc. Med..

[9] Zatońska K., Zatoński W.A., Szuba A. (2016). Prospective urban and rural epidemiology Poland—Study design. J. Health Inequalities.

[10] Polskie Towarzystwo (2019). Diabetologiczne. Zalecenia kliniczne dotyczące postępowania u chorych na cukrzycę 2019. Diabetol. Prakt..

[11] Teo K., Chow C.K., Vaz M., Rangarajan S., Yusuf S., PURE Investigators-Writing Group (2009). The Prospective Urban Rural Epidemiology (PURE) study: Examining the impact of societal influences on chronic noncommunicable diseases in low-, middle-, and high-income countries. Am. Heart J..

[12] Szuba A., Martynowicz H., Zatońska K., Ilow R., Regulska-Ilow B., Różańska D., Wołyniec M., Połtyn-Zaradna K., Zatoński W. (2016). Prevalence of hypertension in Polish population of PURE Poland study. J. Health Inequalities.

[13] World Health Organization Disease Burden and Mortality Estimates. https://www.who.int/data/gho/data/themes/mortality-and-global-health-estimates.

[14] Visseren F.L., Mach F., Smulders Y.M., Carballo D., Koskinas K.C., Back M., Benetos A., Biffi A., Boavida J.M., Capodanno D. (2021). 2021 ESC guidelines on cardiovascular disease prevention in clinical practice. Eur. Heart J..

[15] Livingstone S.J., Looker H.C., Hothersall E.J., Wild S.H., Lindsay R.S., Chalmers J., Cleland S., Leese G.P., McKnight J., Morris A.D. (2012). Risk of cardiovascular disease and total mortality in adults with type 1 diabetes: Scottish registry linkage study. PLoS Med..

[16] Sattar N., Rawshani A., Franzén S., Rawshani A., Svensson A.M., Rosengren A., McGuire D.K., Eliasson B., Gudbjörnsdottir S. (2019). Age at diagnosis of type 2 diabetes mellitus and associations with cardiovascular and mortality risks. Circulation.

[17] Pischon T., Girman C.J., Sacks F.M., Rifai N., Stampfer M.J., Rimm E.B. (2005). Non-high-density lipoprotein cholesterol and apolipoprotein B in the prediction of coronary heart disease in men. Circulation.

[18] Ridker P.M., Rifai N., Cook N.R., Bradwin G., Buring J.E. (2005). Non-HDL cholesterol, apolipoproteins A-I and B100, standard lipid measures, lipid ratios, and CRP as risk factors for cardiovascular disease in women. JAMA.

[19] Larsson S.C., Burgess S. (2022). Appraising the causal role of smoking in multiple diseases: A systematic review and meta-analysis of Mendelian randomization studies. EBioMedicine.

[20] Samson R., Ennezat P.V., Le Jemtel T.H., Oparil S. (2022). Cardiovascular Disease Risk Reduction and Body Mass Index. Curr. Hypertens. Rep..

[21] Whelton S.P., McEvoy J.W., Shaw L., Psaty B.M., Lima J.A., Budoff M., Nasir K., Szklo M., Blumenthal R.S., Blaha M.J. (2020). Association of normal systolic blood pressure level with cardiovascular disease in the absence of risk factors. JAMA Cardiol..

[22] Duarte Lau F., Giugliano R.P. (2022). Lipoprotein(a) and its Significance in Cardiovascular Disease: A Review. JAMA Cardiol..

[23] Klop B., Elte J.W., Cabezas M.C. (2013). Dyslipidemia in obesity: Mechanisms and potential targets. Nutrients.

[24] Pająk A., Szafraniec K., Polak M., Polakowska M., Kozela M., Piotrowski W., Kwaśniewska M., Podolecka E., Kozakiewicz K., Tykarski A. (2016). Changes in the prevalence, treatment, and control of hypercholesterolemia and other dyslipidemias over 10 years in Poland: The WOBASZ study. Pol. Arch. Med. Wewn..

[25] Anza-Ramirez C., Lazo M., Zafra-Tanaka J.H., Avila-Palencia I., Bilal U., Hernández-Vásquez A., Knoll C., Lopez-Olmedo N., Mazariegos M., Moore K. (2022). The urban built environment and adult BMI, obesity, and diabetes in Latin American cities. Nat. Commun..

[26] Kim J., Shon C., Yi S. (2017). The Relationship between Obesity and Urban Environment in Seoul. Int. J. Environ. Res. Public Health.

[27] Carrillo-Larco R.M., Bernabé-Ortiz A., Pillay T.D., Gilman R.H., Sanchez J.F., Poterico J.A., Quispe R., Smeeth L., Miranda J.J. (2016). Obesity risk in rural, urban and rural-to-urban migrants: Prospective results of the PERU MIGRANT study. Int. J. Obes..

[28] Kohli-Lynch C.N., Lewsey J., Boyd K.A., French D.D., Jordan N., Moran A.E., Sattar N., Preiss D., Briggs A.H. (2022). Beyond 10-Year Risk: A Cost-Effectiveness Analysis of Statins for the Primary Prevention of Cardiovascular Disease. Circulation.

[29] Choudhry N.K., Setoguchi S., Levin R., Winkelmayer W.C., Shrank W.H. (2008). Trends in adherence to secondary prevention medications in elderly post-myocardial infarction patients. Pharmacoepidemiol. Drug Saf..

[30] Nieuwkerk P.T., Nierman M.C., Vissers M.N., Locadia M., Greggers-Peusch P., Knape L.P., Kastelein J.J., Sprangers M.A., de Haes H.C., Stroes E.S. (2012). Intervention to improve adherence to lipid-lowering medication and lipid-levels in patients with an increased cardiovascular risk. Am. J. Cardiol..

[31] Haynes R.B., McKibbon K.A., Kanani R. (1996). Systematic review of randomised trials of interventions to assist patients to follow prescriptions for medications. Lancet.

[32] Hagström E., Steg P.G., Szarek M., Bhatt D.L., Bittner V.A., Danchin N., Diaz R., Goodman S.G., Harrington R.A., Jukema J.W. (2022). Apolipoprotein B, Residual Cardiovascular Risk After Acute Coronary Syndrome, and Effects of Alirocumab. Circulation.

[33] Khan S.U., Yedlapati S.H., Lone A.N., Hao Q., Guyatt G., Delvaux N., Bekkering G.E.T., Vandvik P.O., Riaz I.B., Li S. (2022). PCSK9 inhibitors and ezetimibe with or without statin therapy for cardiovascular risk reduction: A systematic review and network meta-analysis. BMJ.

[34] Mihaylova B., Emberson J., Blackwell L., Keech A., Simes J., Barnes E.H., Voysey M., Gray A., Collins R., Baigent C. (2012). The effects of lowering LDL cholesterol with statin therapy in people at low risk of vascular disease: Meta-analysis of individual data from 27 randomised trials. Lancet.

